# Changes in expression of genes related to glucose metabolism in liver and skeletal muscle of rats exposed to acute hypoxia

**DOI:** 10.1016/j.heliyon.2020.e04334

**Published:** 2020-07-02

**Authors:** Yurie Hara, Nakamichi Watanabe

**Affiliations:** a Department of Nutritional Science, Tokyo Kasei University, 1-18-1 Kaga, Itabashi, Tokyo, 173-8602, Japan; b Department of Health Science, Showa Women's University, 1-7-57 Taishido, Setagaya, Tokyo, 154-8533, Japan

**Keywords:** Biochemistry, Molecular biology, Physiology, Organ system, Glycolysis, Normobaric hypoxia, Glycogen, Gluconeogenesis

## Abstract

The aim of this study was to determine changes in gene expression associated with glucose metabolism in the liver and soleus muscles of rats exposed to hypoxia to improve work capacity under high altitude conditions. Rats were divided into normobaric normoxia (control) and normobaric hypoxia (hypoxia) groups (*n* = 7 each), and the hypoxia group was exposed to 10.5% oxygen for 90 min. Glucose metabolism-related gene expression was examined by real-time polymerase chain reaction. In the liver, the expression levels of the glucose utilization-related genes solute carrier family 2 member 1, glucokinase, and liver-type phosphofructokinase and the gluconeogenesis-related gene phosphoenolpyruvate carboxykinase 1 (*Pck1*) were significantly increased upon hypoxic exposure. In contrast, gene expression in the soleus was unchanged, with the exception of *Pck1*. The results suggest that under hypoxia, both glucose utilization and gluconeogenesis are accelerated in the liver, and liver glycogen is degraded to maintain blood glucose level.

## 1 Introduction

Hypoxic conditions, such as high altitudes and strenuous exercise, induce metabolic changes [1]. For example, systemic glucose utilization is accelerated in both humans and rats under hypoxic conditions [2, 3, 4], and glycogen degradation in the liver is crucial to maintain blood glucose levels under these conditions. In fact, hypoxic exposure reduces liver glycogen contents in rats and mice [3, 5, 6, 7, 8]. These metabolic changes, i.e., the acceleration of glucose utilization and the depletion of liver glycogen, induce hypoglycemia, which can result in decreased work capacity due to increased fatigue [9, 10]. To mitigate the effects of hypoxia on work capacity, understanding glucose metabolism changes in the liver, the main metabolic organ, and the skeletal muscle, the largest working organ, is essential.

However, two important questions remain unanswered. First, the organs in which glucose utilization is accelerated under hypoxic conditions are unknown. Second, the role of glycogen metabolism in hypoxia remains unclear, with contradictory observations reported [3, 5, 6, 7, 8, 11, 12, 13]. For example, hypoxic exposure has been shown to increase glycogen accumulation induced by glycogen synthase activation in myoblasts, ovarian clear cell carcinoma, and cultured rat heart muscle [11, 12, 13].

To better understand glucose metabolism changes under hypoxia, we have exposed rats to hypoxia and examined changes in the expression of genes related to glucose metabolism in the livers and soleus muscles of hypoxic rats. As mRNA levels can reflect protein expression and phenotype [14, 15, 16] in hypoxia, we used real-time reverse transcription-polymerase chain reaction (RT-PCR).

## 2 Materials and methods

### 2.1 Animals and experimental protocol

Eight-week-old male Sprague-Dawley rats were purchased from Charles River Laboratories (Yokohama, Japan). The rats were individually maintained in plastic cages at 21 ± 2 °C with a 12-h light-dark cycle (light time: 6:00–18:00) and fed a commercial purified chow diet (CRF-1; Charles River Laboratories). After acclimatization for 1 week, the rats were divided into normobaric normoxia (control) and normobaric hypoxia (hypoxia) groups (*n* = 7/group). After fasting overnight to diminish the effects of feeding, the hypoxia group was exposed to hypoxia in a sealed hypoxic box, while the control group was placed in a similar uncovered box. Hypoxic conditions, namely, the oxygen concentration and the exposure duration, were optimized at the beginning of the study. Oxygen concentration was maintained at 10.5%, which is recognized as moderate hypoxia in animal models [17]. The hypoxic exposure duration was set to 90 min, as gene expression changes have been reported 80 min after hypoxic exposure [18]. The oxygen concentration of the hypoxia group was first decreased from 21.0% to 10.5% for 30 min by gradually adjusting the mixture of oxygen and nitrogen, and then maintained at 10.5% for 90 min. The respiration rate was measured visually once at 21.0, 17.5, 15.0, and 10.5% oxygen, i.e., at the 0, 10, 20, and 30 min time points of the gradual oxygen reduction. Oxygen concentrations were measured using an OXYMAN OM-25MF10 Oxygen Monitor (Taiei Engineering Co., Ibaraki, Japan). After hypoxic exposure, the rats were sacrificed and whole blood collected from the abdominal aorta under isoflurane anesthesia. The collected blood was centrifuged at 1,900 × *g* for 10 min to obtain plasma, which was stored at −80 °C until the free fatty acid content was measured. The liver and soleus muscle were stored at −80 °C until the glycogen content was measured. Section of the liver and soleus muscle were immersed in RNAlater Stabilization Solution (Qiagen, Hilden, Germany) and stored according to the manufacturer's instructions. All animal experiments were performed according to a research protocol approved by Showa Women's University (approval number: 14-03).

### 2.2 Measurement of blood glucose, blood lactate, and plasma free fatty acid levels

Blood glucose and blood lactate levels were measured in duplicate at the time of dissection using Medisafe Chips (Terumo, Tokyo, Japan) and a Lactate Pro Blood Lactate Meter (Arkray, Kyoto, Japan), respectively. Plasma free fatty acid levels were measured using the NEFA C test kit (Wako Pure Chemical Industries, Ltd., Osaka, Japan) according to the manufacturer's instructions.

### 2.3 Measurement of glycogen contents in the liver and soleus muscle

Glycogen contents in the liver and soleus muscle were measured using the phenol-sulfuric acid method. First, 50 or 100 mg of liver or soleus muscle, respectively, were homogenized in 0.8 mL of 10% trichloroacetic acid (Wako Pure Chemical Industries Ltd.), followed by centrifugation at 1,900 × *g* for 10 min for deproteinization. The supernatant (0.4 mL) was mixed with 0.8% ethanol (Wako Pure Chemical Industries Ltd.), followed by centrifugation at 1,900 × *g* for 10 min to precipitate glycogen. Then, the supernatant was decanted and the precipitated glycogen was r-suspended in 0.5 mL distilled water. Phenol (0.5 mL of 5% stock; Wako Pure Chemical Industries Ltd.) and 2.5 mL concentrated sulfuric acid (Kanto Chemical Co., Inc., Tokyo, Japan) were added to the resuspended glycogen solution, and the mixture was incubated for 20 min at room temperature (20–22 °C). Then, the absorbance was measured at 490 nm using a U-5100 spectrophotometer (Hitachi High-Tech Science Co., Tokyo, Japan). A standard curve was generated using a 40 mg/dL glucose (Wako Pure Chemical Industries Ltd.) solution.

### 2.4 mRNA expression in the liver and soleus muscle

Total RNA was isolated from livers and soleus muscles immersed in RNAlater using the RNeasy Mini Kit (Qiagen) and RNeasy Fibrous Tissue Mini Kit (Qiagen), respectively, according to the manufacturer's instructions. Reverse transcription was performed using the PrimeScript RT Reagent Kit (Perfect Real Time; TaKaRa Bio, Shiga, Japan) according to the manufacturer's instructions, and products were stored at −80 °C. RT-PCR was performed using Power SYBR Green PCR Master Mix (Applied Biosystems, Foster City, CA, USA) and an Applied Biosystems 7500 RT-PCR system. The reaction conditions were as follows: denaturation at 95 °C for 10 min; then 40 cycles of denaturation at 95 °C for 15 s and annealing/extension at 60 °C for 1 min. Target genes and primer sequences are shown in Table 1. Target gene expression was determined using the 2^ΔΔCt^ method [19] and β-actin was used as an internal control.

**Table 1:** Primer sequences for real-teime PCR.

Gene name	Gene symbol	Forward Primers (5′ to 3′)	Reverse Primers (5′ to 3′)
Liver			
Glucose uptake			
Solute carrier family 2 (facilitated glucose transporter), member 1 (*Slc2a1*)	*Slc2a1*	GACCCTGCATCTGATTGGTCTG	CCACAATGAACCATGGAATAGGA
Solute carrier family 2 (facilitated glucose transporter), member 2 (*Slc2a2*)	*Slc2a2*	TTGGTGCCATCAACATGATCTTC	AGATGGCCGTCATGCTCACATA
Glycolysis			
Glucokinase	*Gck*	AGTATGACCGGATGGTGGATGAA	CCAGCTTAAGCAGCACAAGTCGTA
Phosphofructokinase	*Pfkl*	CCACCTGGAGGCCATTGATGA	GGGATGACGCACATGACGA
Pyruvate kinase	*Pklr*	ATCTGGGCAGATGATGTGGA	ATAGGGTGTAACTGGGTCAGAATGG
Glycogen metabolism			
Glycogen synthase 2	*Gys2*	CATGAATGGCAGGCTGGAAC	GCTCCATGCAGTAGCGGTGA
Glycogen phosphorylase	*Pygl*	GATCCGCACACAGCAGCACTA	CTTCGTCGCAGGCATTCTGTAA
Gluconeogenesis			
Glucose-6-phosphatase	*G6pc*	TTAGAGGCAAAGGAGCCCAAG	GGGTGGAAACACAGGCATCA
Phosphoenolpyruvate carboxykinase 1	*Pck1*	CAGCCAATGTCCCATTATTGACC	TGCCAGCTGAGAGCTTCGTAGA

Soleus muscle			
Glucose uptake			
Solute carrier family 4 (facilitated glucose transporter), member 4 (*Slc2a4*)	*Slc2a4*	CTCCAACTGGACCTGTAACTTCATC	GCCTCTGGTTTCAGGCACTC
Glycolysis			
Hexokinase 1	*Hk1*	ATTGTCGCCGTGGTGAATGA	TAGCAAGCATTGGTGCCTGTG
Hexokinase 2	*Hk2*	TCGATGGCTCCGTCTACAAGAA	ACATCACAGTCGGGCACCAG
Phosphofructokinase	*Pfkm*	GGGCTGACACAGCACTGAACA	GGCCAGATAGCCACAGTAACCAC
Pyruvate kinase	*Pkm*	TGTTTAGCAGCAGCTTTGATAGTTC	GCGTGTCACAGCAATGATAGGAG
Glycogen metabolism			
Glycogen synthase 1	*Gys1*	TCAGAGCAAAGCACGAATCCAG	AACTCATAGCGTCCAGCGATAAAGA
Glycogen phosphorylase	*Pygm*	TCCGCACACAGCAGCATTACTAC	TCCAAGGCCAGGTTCACCA
Gluconeogenesis			
Phosphoenolpyruvate carboxykinase 1	*Pck1*	CAGCCAATGTCCCATTATTGACC	TGCCAGCTGAGAGCTTCGTAGA

### 2.5 Statistical analysis

All data are expressed as the mean ± standard error (SE). Statistically significant differences (*p* < 0.05) between the control and hypoxia groups were evaluated using Student's *t*-test. Statistical significant differences in respiration rate in the hypoxia group between 21.0% oxygen and lower concentrations were evaluated using Dunnett's test. Statistical analysis was performed using GraphPad Prism 7 (GraphPad Software, La Jolla, CA, USA).

## 3 Results

### 3.1 Respiration rate

The respiration rate of the control group (measured visually) was 84 ± 3 breaths/min, while those of the hypoxia group were 96 ± 5, 114 ± 10, 119 ± 8, and 168 ± 7 times/min at 21.0, 17.5, 15.0, and 10.5% O_2_, respectively. Significant increases in respiration rate were observed at 15.0% and 10.5% O_2_ compared to 21.0%.

### 3.2 Blood glucose, blood lactate, and plasma free fatty acid levels

The levels of blood glucose, blood lactate, and plasma free fatty acids are shown in Table 2. The blood glucose and plasma free fatty acid levels of the control and hypoxia groups were not significantly different, while the blood lactate level of the hypoxia group was significantly lower than that of the control group.

**Table 2:** The levels of blood glucose, blood lactate, plasma free fatty acid, liver glycogen, and soleus muscle glycogen.

	Control	Hypoxia
Blood glucose (mg/dL)	139 ± 7	136 ± 6
Blood lactate (mM)	2.0 ± 0.1	1.4 ± 0.1∗∗
Plasma free fatty acid (mEq/dL)	0.75 ± 0.15	0.69 ± 0.14
Liver glycogen (mg/g)	42.4 ± 6.4	26.6 ± 5.1
Soleus muscle glycogen (mg/g)	8.4 ± 0.4	8.9 ± 0.4

Data are mean ± SE in 7 rats.

∗∗Significantly different from the control group: p < 0.01.

### 3.3 Glycogen contents of the liver and soleus muscle

The glycogen contents of the liver and soleus muscle are shown in Table 2. Although the liver glycogen content in the hypoxia group was 37% lower than that in the control group, the difference was not statistically significant (*p* = 0.09). Soleus muscle glycogen content in the control and hypoxia groups were not significantly different.

### 3.4 Liver mRNA expression

**Figure 1:**
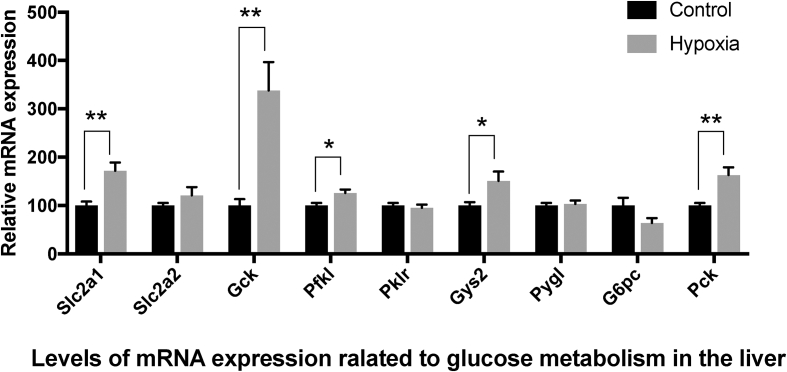
mRNA expression of genes related to glucose metabolism in the liver. Data represent mean ± SE of 7 rats. Values are significantly different from that of the control group at ∗P < 0.05 and ∗∗P < 0.01.

The relative mRNA expression levels of glucose metabolism-related genes in the liver are shown in Figure 1. Among genes required for glucose uptake, the expression of solute carrier family 2 member 1 (*Slc2a1*) was 1.7-fold higher in the hypoxia group than in the control group (*p* = 0.002). Although the expression of solute carrier family 2 member 2 (*Slc2a2*) was 1.2-fold higher in the hypoxia group compared to the control group, the difference was not statistically significant. Of the three genes encoding rate-limiting glycolytic enzymes (glucokinase (*Gck*), phosphofructokinase liver type (*Pfkl*), and pyruvate kinase L/R (*Pklr*), the levels of *Gck* and *Pfkl* were 3.4-fold and 1.3-fold higher (*p* = 0.002 and 0.011, respectively) in the hypoxia group compared to the control group, while *Pklr* expression was not significantly different between the two groups. Of the genes encoding enzymes required for glycogen metabolism, the expression of glycogen synthase 2 (*Gys2*) was 1.5-fold higher in the hypoxia group than in the control group (*p* = 0.027), while glycogen phosphorylase (*Pygl*) was not significantly different. Among genes encoding enzymes required for gluconeogenesis, the expression of phosphoenolpyruvate carboxykinase 1 (*Pck1*), a rate-limiting gluconeogenesis enzyme in the liver, was 1.6-fold higher in the hypoxia group than in the control group (*p* = 0.003), while glucose-6-phosphatase (*G6pc*) did not significantly differ between the groups.

### 3.5 Soleus muscle mRNA expression

**Figure 2:**
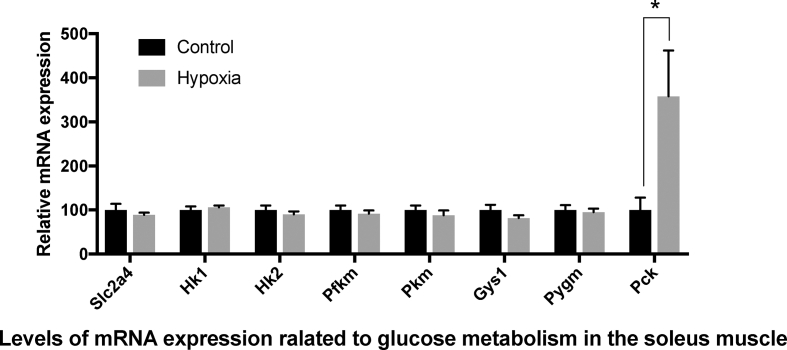
mRNA expression of genes related to glucose metabolism in the soleus muscle. Data represent mean ± SE of 7 rats. Values are significantly different from that of the control group at ∗P < 0.05.

The relative mRNA levels of glucose metabolism-related genes in the soleus muscle are shown in Figure 2. Unlike in the liver, the expression levels of genes related to glucose uptake, glycolysis, and glycogen metabolism were not significantly different between the hypoxia and control groups. The only exception was *Pck*, which displayed 3.6-fold higher expression in the hypoxia group compared to the control group (*p* = 0.034).

## 4 Discussion

The aim of this study was to examine changes in the expression of genes related to glucose metabolism in the liver and soleus muscle of rats exposed to normobaric hypoxia, to identify organs with accelerated glucose utilization in this condition and assess changes in liver glycogen content. This study suggests that hypoxic exposure enhances the expression of genes related to glucose uptake (*Slc2a1)* and glycolysis (*Gck* and *Pfkl*) in the liver, while glucose metabolism-related gene expression in the soleus muscle was not affected. This indicates that hypoxic exposure may specifically decrease glycogen content in the liver. Furthermore, the levels of gluconeogenesis- and glucose utilization-related genes were also promoted in the liver.

The acceleration of glucose uptake and glycolysis under hypoxia is crucial to compensate for decreased mitochondrial ATP synthesis via the tricarboxylic acid cycle and β-oxidation. Hypoxia has been reported to accelerate glucose uptake and glycolysis in the body [2, 4, 20, 21]. The results of these systemic studies were consistent with those observed in the liver in this study, with increased *Slc2a1*, *Gck*, and *Pfkl* expression. These genes were not overexpressed in the soleus muscle. As *Slc2a1*, *Gck*, and *Pfkl* are rate-limiting glycolytic enzymes, the results suggest that the liver is one of the main organs where glucose utilization is accelerated under hypoxia.

Accelerated glucose utilization under hypoxia may induce hypoglycemia, and degradation of liver glycogen plays an important role in preventing this [22]. For instance, the liver glycogen level in rats decreased by 53% after exposure to 5% O_2_ for 30 min [3]. In another report, the liver glycogen level decreased by 50% when mice were exposed to an altitude of 3,800 m for 30 days [5]. Here, we observed that hypoxic exposure reduced liver glycogen content by 37%, although the difference was not statistically significant. Because this study was performed in fasting rats, the initial liver glycogen levels were likely much lower than in fed rats. Hypoxia may induce a more significant decrease in liver glycogen in fed rats. We measured gene expression levels to determine the mechanism underlying the decreased glycogen content, and observed that *Gys2* expression increased significantly after hypoxic exposure while *Pygl* expression was unchanged. The increase in *Gys2* expression might have been due to a homeostatic response to the decreased liver glycogen content under hypoxia. Indeed, hypoxia increases the expression and activity of glycogen synthase in myoblast cells [12]. Therefore, we postulate that decreased liver glycogen content is important to maintain the blood glucose level under hypoxia.

We observed that hypoxic exposure significantly increased the expression of liver PCK1, the rate-limiting enzyme of gluconeogenesis. As gluconeogenesis is regulated by PCK levels in the liver [23, 24, 25], the results suggest that hypoxic exposure accelerates gluconeogenesis. The blood level of lactate, an important substrate for gluconeogenesis, decreased significantly under hypoxia. Although hypoxia generally increases the blood lactate level, an activated hypoxic response in prolyl hydroxylase-knockout mice accelerated glucose uptake in the liver by activating the Cori cycle, which subsequently decreased the blood lactate level [26]. Therefore, to maintain the blood glucose level, the response to hypoxic exposure may involve increasing not only liver glycogen degradation but also gluconeogenesis. Although hypoxic exposure is known to increase *Pck1* expression in adipose tissue [27], to our knowledge, this is the first study demonstrating hypoxia-induced upregulation of *Pck1* in the liver, the major site of gluconeogenesis. However, as mentioned above, this study was performed in fasting rats, and liver glycogen stores may have been depleted. Therefore, it is possible that our results underestimate the contributions of liver gluconeogenesis in response to hypoxia.

While hypoxic exposure altered the expression of various glucose metabolism-related genes in rat livers, the expression of these genes in the soleus was unaffected, except for *Pck1*. This can be explained as follows: while the metabolism of the liver, a vital organ, must adapt to hypoxic conditions, skeletal muscle activity was reduced in hypoxia, as the rats stopped moving in the presence of 15% oxygen and assumed a prone position in 10.5% oxygen. In fact, in the cardiac muscle of rats exercised in hypoxic conditions, solute carrier family 2 member 4 expression actually increased [28, 29]. The role of the marked increase in *Pck1* expression in the soleus muscle is unclear, although its overexpression in mouse skeletal muscle results in a marked increase in physical activity [30]. Therefore, examining gene expression changes in an exercised rat model under hypoxic conditions will be required to fully understand the metabolic changes in skeletal muscle under hypoxia.

## 5 Conclusion

Gene expression analyses in the livers and soleus muscles of rats exposed to hypoxia demonstrated that the liver is one of the major organs where glucose utilization is accelerated under hypoxia. Glycogen degradation and gluconeogenesis were also accelerated in the liver under hypoxia to help maintain blood glucose levels with increased utilization.

## Declarations

### Author contribution statement

Yurie Hara: Conceived and designed the experiments; Performed the experiments; Analyzed and interpreted the data; Wrote the paper.

Nakamichi Watanabe: Conceived and designed the experiments; Analyzed and interpreted the data; Wrote the paper.

### Funding statement

This work was supported by 10.13039/501100001691JSPS KAKENHI grant number JP 25750054.

### Competing interest statement

The authors declare no conflict of interest.

### Additional information

No additional information is available for this paper.
